# How the intricate relationship between nutrition and hormonal equilibrium significantly influences endocrine and reproductive health in adolescent girls

**DOI:** 10.3389/fnut.2024.1337328

**Published:** 2024-03-14

**Authors:** Valeria Calcaterra, Elvira Verduci, Stefano Stagi, Gianvincenzo Zuccotti

**Affiliations:** ^1^Department of Internal Medicine and Therapeutics, University of Pavia, Pavia, Italy; ^2^Department of Pediatric, Buzzi Children's Hospital, Milan, Italy; ^3^Department of Health Sciences, University of Milan, Milan, Italy; ^4^Department of Health Sciences, University of Florence, Florence, Italy; ^5^Meyer Children's Hospital Scientific Institute for Research, Hospitalization and Health Care (IRCCS), Florence, Italy; ^6^Department of Biomedical and Clinical Science, University of Milan, Milan, Italy

**Keywords:** nutrition, hormones, reproductive health, endocrine system, fertility, adolescents

Nutrition is emerging as a pivotal environmental factor shaping both the endocrinological landscape and reproductive capabilities (1–3).

Through their role as precursors to crucial molecules involved in bodily reactions, nutrients wield significant influence over physiological processes and biochemical pathways, including those governing hormones (4). Maintaining hormonal balance is crucial for sustaining reproductive functions and fertility.

Thus, the quantity, quality, and composition of foods wield substantial influence over our health span, particularly impacting endocrine and reproductive functions from intrauterine development through adolescence (5, 6).

In this opinion paper, we aim to underscore the profound interplay between food and hormones and its ramifications on endocrine and reproductive health, a relationship evident from fetal inception to adulthood. Sharing viewpoints fosters discourse and prompts reflection on strategies to safeguard reproductive wellbeing.

Nutrition during the periconceptional period and pregnancy emerges as a critical determinant for fetal development, growth, and overall wellbeing. Inadequate nutrition can disrupt the normal development and growth of organs, including the hypothalamic-pituitary-gonadal axis (HPA-axis), thereby potentially affecting endocrine, reproductive health, and fertility in adulthood (7).

The reproductive capability is influenced by maternal nutritional status through various pathways.

Firstly, nutrition during gestation can influence the development of the fetal endocrine and reproductive systems (8). The transportation of nutrients and metabolic processes within the placenta play a vital role in determining fetal weight, which represents a crucial factor in the programming of endocrine systems during critical phases of fetal development (6). As reported in the literature (6), restricted prenatal growth may lead to permanent alterations in endocrine axes, subsequently affecting sexual maturation and reproductive function. A deficiency in key nutrients during this critical period may lead to structural and functional changes in organs, including the hypothalamus, pituitary gland, thyroid and gonads; these changes can affect numerous aspects of growth and development, potentially causing hormonal imbalances and infertility (7). Additionally, the theory of “metabolic programming” suggests that specific nutritional conditions during fetal development can influence metabolism and health in adulthood, increasing the risk of developing metabolic diseases like type 2 diabetes and obesity (9), which, in turn, can affect endocrinological balance and fertility (Figure 1).

**Figure 1:**
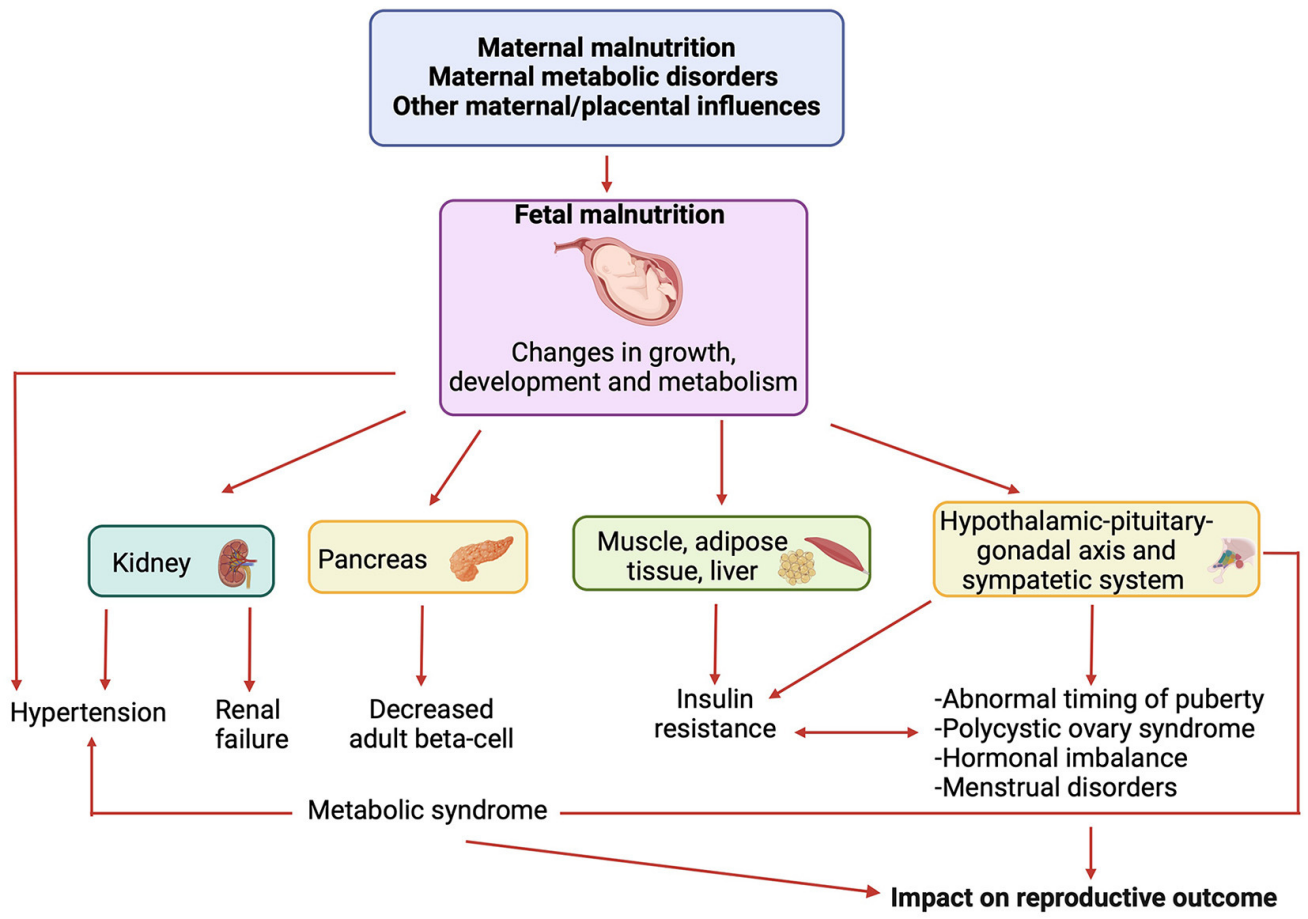
Link between nutritional conditions during fetal development, metabolic diseases and reproductive outcome. Created by Biorender.com.

In addition, nutrition profoundly impacts the quality of oocytes, with deficiencies in antioxidants, vitamins, and minerals during pregnancy potentially leading to transgenerational consequences (2, 10).

Reproductive outcomes are also influenced by maternal metabolic status during pregnancy, with gestational diabetes mellitus (GDM) associated not only with adverse fetal outcomes, such as death from birth, visceromegaly, and fetal macrosomia but also a heightened risk of metabolic disorders in children (11), such as hyperinsulinism and type 2 diabetes. These disorders may impact the timing of pubertal development and conditions such as polycystic ovary syndrome (PCOS), thereby influencing women's reproductive health (12) (Figure 1).

Childhood and adolescence are critical periods for protecting endocrine and reproductive health, with nutrition playing a significant role in all stages of growth, particularly during pubertal development (6).

Puberty represents a crucial phrase of physical maturation, marked by the activation of the hypothalamic-pituitary-gonadal (HPG) axis and the release of hormones like gonadotropin-releasing hormone (GnRH), follicle-stimulating hormone (FSH), and luteinizing hormone (LH). Both genetic and environmental factors are involved in the activation and maintenance of HPG integrity. Adequate nutrition is essential for the initiation and progression of puberty, as nutrients serve as cofactors, precursors, and regulators in the synthesis and function of reproductive hormones, such as gonadotropin and sex hormones such as estrogen, progesterone, and testosterone. Thus, proper nutrition is linked to consistent hormonal production, and consequently, plays a crucial role in the development of secondary sexual characteristics and menstrual cycles (6).

The onset of puberty requires a state of positive energy balance, with chronic malnutrition potentially delaying puberty (13) and overnutrition and obesity which can be associated with increased estrogen production and leptin levels, contributing to early puberty (14).

Different nutritional choices have been associated with distinct patterns of puberty, with diets high in energy, fat, and protein, and a high glycemic index linked to unbalanced micronutrient supplies involved in hormonal stimulation, leading to precocious puberty (15); proposed pathogenic mechanisms implicated in this early activation include the activation of GnRH via hypothalamic inflammation and microglial cell activation, dietary signals affecting the hypothalamus, alterations in gut microbiota influencing hormone secretion, and the overexpression of transcription factors (15).

Disruptions in normal pubertal development may have long term health effects including menstrual irregularities and infertility in adulthood (16). Early pubertal maturation, such as premature adrenarche and premature pubarche, represents childhood risk factors for PCOS, impacting reproductive function (12).

Nutritional imbalance and dietary patterns can disrupt the normal development and functioning of the reproductive system, affecting the regularity of ovulation and the quality of oocytes released during the menstrual cycle. Diets rich in fish and seafood, vegetables and fruit, cereals, and low-fat dairy products are positively correlated with the quality of ovulation (2). On the contrary, diets rich in processed meats, soy, potatoes, full-fat dairy products, sugary drinks, and sweets seem to negatively impact endocrine and reproductive health (2, 17).

Obesity, in particular, poses significant challenges to reproductive function, with obese adolescents and women at increased risk of menstrual dysfunction, anovulation, PCOS, subfecundity, and infertility (14, 18). Female obesity disrupts the HPG axis, leading to hormonal imbalances that impair reproductive control (14).

This arises from heightened peripheral conversion of androgens to estrogens, hyperandrogenism stemming from insulin resistance and hyperinsulinemia, thyroid irregularities, reduced levels of sex hormone-binding globulin, growth hormone, and insulin-like growth factor binding proteins, alongside elevated leptin levels. These collective hormonal disturbances contribute to impaired regulation of the reproductive system (14). Specifically, hyperandrogenism and insulin resistance are key factors in the etiology of PCOS (19), which often emerges during the early puberty and affects pregnancy outcomes, such as an increased risk of gestational diabetes mellitus (GDM), pregnancy-induced hypertension, and preeclampsia (19).

Chronic inflammation and oxidative stress associated with overnutrition can further negatively impact fertility (20, 21). Additionally, metabolic disorders linked to obesity, such as insulin resistance and hyperandrogenism, can disrupt hormonal balance and contribute to conditions like PCOS, thus impacting fertility (14).

Similar to obesity, underweight individuals may also face challenges associated with the regulation of the endocrine system, including the HPG- and growth hormone—insulin-like growth factor (IGF)-1 axis, thyroid and adrenal functions. These factors can influence pubertal development, sexual characteristics, and fertility (22, 23).

In conclusion, the intricate relationship between nutrition and hormonal balance profoundly impacts female endocrine and reproductive health. From fetal development onward, nutritional, and metabolic factors significantly influence reproductive outcomes. Recognizing nutrition as a modifiable factor in preventing adverse reproductive outcomes is crucial. A balanced diet rich in essential nutrients plays a pivotal role in maintaining hormonal equilibrium, preventing reproductive disorders, and safeguarding fertility. Embracing a holistic approach that integrates nutrition with healthy lifestyle practices is instrumental in promoting the reproductive wellbeing of adolescent girls.

## Author contributions

VC: Conceptualization, Supervision, Writing—original draft, Writing—review & editing. EV: Conceptualization, Supervision, Writing—original draft, Writing—review & editing. SS: Conceptualization, Supervision, Writing—original draft, Writing—review & editing. GZ: Conceptualization, Supervision, Writing—original draft, Writing—review & editing.
